# A uniformly valid approximation algorithm for nonlinear ordinary singular perturbation problems with boundary layer solutions

**DOI:** 10.1186/s40064-016-1865-6

**Published:** 2016-03-05

**Authors:** Süleyman Cengizci, Mehmet Tarık Atay, Aytekin Eryılmaz

**Affiliations:** Institute of Applied Mathematics, Middle East Technical University, 06800 Ankara, Turkey; Department of Mechanical Engineering, Abdullah Gül University, 38039 Kayseri, Turkey; Department of Mathematics, Nevşehir Hacı Bektaş Veli University, 50300 Nevşehir, Turkey

**Keywords:** Uniformly valid approximation, Asymptotic expansion, Boundary layer, Singular perturbation, Successive Complementary Expansion Method, 34B15, 34E15, 65L15

## Abstract

This paper is concerned with two-point boundary value problems for singularly perturbed nonlinear ordinary differential equations. The case when the solution only has one boundary layer is examined. An efficient method so called Successive Complementary Expansion Method (SCEM) is used to obtain uniformly valid approximations to this kind of solutions. Four test problems are considered to check the efficiency and accuracy of the proposed method. The numerical results are found in good agreement with exact and existing solutions in literature. The results confirm that SCEM has a superiority over other existing methods in terms of easy-applicability and effectiveness.

## Background

Nonlinear problems have always been more attractive than linear ones for scientists. The main reason for this is that almost all natural phenomena in nature lead us to nonlinear models to describe them. In these models, finding exact solutions are quite difficult or sometimes impossible. Because of this reason, it is needed to get at least an approximate solution to these types of problems by certain methods.

Singularly perturbed problems occurs frequently in electrical systems, celestial mechanics, particle physics, quantum mechanics, (semi/super) conductor systems, fluid mechanics, thermal processes and in chemical/biochemical reactions (Kumar 2011). These problems are characterized by the presence of a very small positive parameter $0< \varepsilon \ll 1$ that multiplies the highest order derivative term in the differential equation. This small parameter is known as *singular perturbation parameter*. In the case of $\varepsilon =0$, problem is called as *reduced problem* since the order of the differential equation reduces. Consider the general form of a singularly perturbed nonlinear second-order ordinary differential equation

1 $$\begin{aligned} \varepsilon y^{\prime \prime }(x)=f(x,y(x),y^{\prime }(x)) \end{aligned}$$

subject to boundary condions

2 $$\begin{aligned} y(0)=\alpha , ~y(1)=\beta , \quad ~(\alpha ,\beta \in {\mathbb {R}}). \end{aligned}$$

It is a well established fact that a nonlinear second-order two-point boundary value problem (1–2) with small parameter plays a critical role in nonlinear physics. Also it is of great practical interest to study this nonlinear phenomena. Singular perturbation problems and the methods used to tackle them are very important concepts because of their mathematical properties, physical meanings and applications in engineering sciences. We refer the reader to consult the reference Kumar (2011) for more detailed information and some significant examples.

The first study in the perturbation theory was presented by Prandtl (1905). But, the term *singular perturbation* was used for the first time by Friedrichs and Wasow (1946). Scientists have paid great attention for this theory for more than a century. After the first studies, a number of excellent books were published such as O’Malley (1974), Bender and Orszag (1978), Kevorkian and Cole (1981), Eckhaus (1973), Eckhaus (1979), Lagerstrom (1988), Hinch (1991), Van Dyke (1975), Johnson (2006), Verhulst (2006), Holmes (1995) and Roos et al. (1996). Thanks to these great books and the other works, today we have certain *traditional asymptotic methods*. Some of them are the Method of Matched Asymptotic Expansions (MMAE), the Method of Multiple-scale Analysis, the Periodic Averaging Method, the Method of Wentzel–Kramers–Brillouin (WKB) Approximation and the Method of Strained Coordinates.

Towards the end of 1980s, various methods, apart from the traditional asymptotic methods, began to appear. In those years Kadalbajoo, Reddy, Jiwari et al. conducted so many significant studies such as Kadalbajoo et al. (1987), Kadalbajoo and Reddy (1987), Chawla and Katti (1982), Mo (1993), Kadalbajoo and Patidar (2003), Kadalbajoo and Gupta (2010), Mittal and Jiwari (2011), Sharma et al. (2012a) and Sharma et al. (2012b). In 2003, Kadalbajoo and Patidar made a detailed survey of singular perturbation problems in partial differential equations (PDEs) (Kadalbajoo and Patidar 2003). In 2010, Kadalbajoo and Gupta in their study Kadalbajoo and Gupta (2010) made a great survey on the numerical methods for singularly perturbed problems. In 2011, Parul studied the traditional methods to solve this kind of problems and gave important examples occuring in engineering and science (Kumar 2011, 2011). In 2012, Roos made a survey, particularly of singularly perturbed convection–reaction–diffusion problems covering the years 2008–2012 (Roos 2012). In the mean time, a number of intriguing numerical methods were presented such as Reproducing Kernel Method (Cui and Geng 2007; Geng and Cui 2007; Geng 2011; Geng and Cui 2011; Li et al. 2012), Variational Iteration Method (Kumar and Mishra 2014), Haar Wavelet Approach (Pandit and Kumar 2014). We must state that there are so many various methods and the above-mentioned methods are just some of them.

In this paper, we study on an efficient asymptotic method called SCEM that generates uniformly valid approximations (UVA) to the solution of singularly perturbed nonlinear boundary value problems. Applying the present method, we are able to get rid of tedious matching procedure of MMAE. We propose a UVA at the first step and then seek appropriate approximations called outer approximation and complementary approximations such that the resulting successive approximations satisfy the boundary conditions exactly.

The paper is organized as follows: “About asymptotic expansions” section gives a brief description of the asymptotic expansions. The overview of SCEM is given in “Successive Complementary Expansion Method” section. In “Numerical examples” section, we consider four numerical problems for comparison with existing methods. The conclusion is given in the last section.

## About asymptotic expansions

In this section, we briefly review the basic concepts of asymptotic approximation theory.

The limit process is fundamental tool of mathematical analysis. It is defined for a given real continuous function *f*: given any $$\delta >0$$ there exists a number $$n_{0}\left( \delta \right)$$ such that $$\left| f\left( x\right) -f\right| <\delta$$ for any $$n\eqslantgtr n_{0}\left( \delta \right) .$$ This definition gives information about the behavior of the function *f* as $$x\rightarrow a,$$ but not about how. Therefore, asymptotic approximation theory requires another definition that enables us to describe the behavior of functions and compare them in a more precise way under the limit process: Bachmann–Landau notations. Consider real and continuous functions $$f\left( \varepsilon \right)$$ and $$g\left( \varepsilon \right) ,$$ where $$0<\varepsilon \le \varepsilon _{0}\ll 1.$$$$f\left( \varepsilon \right) =O\left( g\left( \varepsilon \right) \right) ,$$$$\varepsilon \rightarrow 0$$ if there exist positive constants *K* and $$\varepsilon _{0}$$ such that $$\left| f\left( \varepsilon \right) \right| \le K\left| g\left( \varepsilon \right) \right|$$ for $$\varepsilon \rightarrow 0$$ in $$\left( 0,\varepsilon _{0}\right] .$$$$f\left( \varepsilon \right) =o\left( g\left( \varepsilon \right) \right) ,$$ for $$\varepsilon \rightarrow 0$$ if $${\lim\nolimits_{\varepsilon \rightarrow 0}}\frac{f\left( \varepsilon \right) }{g\left( \varepsilon \right) }=0$$. $$f\left( \varepsilon \right)$$ and $$g\left( \varepsilon \right)$$ are said to be asymptotically equivalent, $$f\left( \varepsilon \right) \approx g\left( \varepsilon \right)$$ as $$\varepsilon \rightarrow 0$$ if $$f\left( \varepsilon \right) =O\left( g\left( \varepsilon \right) \right)$$ and $$g\left( \varepsilon \right) =O\left( f\left( \varepsilon \right) \right)$$ for $$\varepsilon \rightarrow 0$$. $$f\left( \varepsilon \right) =O_{S}\left( g\left( \varepsilon \right) \right)$$ if $$f\left( \varepsilon \right) =O\left( g\left( \varepsilon \right) \right)$$ and $$f\left( \varepsilon \right) \ne o\left( g\left( \varepsilon \right) \right)$$ for $$\varepsilon \rightarrow 0$$. Here the subscript *S* denotes *sharp estimate*. Now consider a sequence of functions $$\left\{ \phi _{n+1}\right\}$$, $$n=0,1, \ldots$$ . Such a sequence is an *asymptotic sequence* if $$\phi _{n+1}\left( \varepsilon \right) =o\left( \phi _{n}\left( \varepsilon \right) \right)$$ for $$\varepsilon \rightarrow 0$$ and $$n=0,1, \ldots$$ Let $$y\left( x,\varepsilon \right)$$ be defined in some domain $$\Omega$$ of *x* and some neighborhood of $$\varepsilon =0$$. The series

3 $$\begin{aligned} y_{a}\left( x,\varepsilon \right) =\sum \limits _{i=0}^{n}\phi _{i}\left( \varepsilon \right) y_{i}\left( x\right) \end{aligned}$$

is called *regular asymptotic expansion (Poincaré expansion)* of $y\left( x,\varepsilon \right)$ as $\varepsilon \rightarrow 0$ if the condition

$$\begin{aligned} y\left( x,\varepsilon \right) -\sum \limits _{i=0}^{n}\phi _{i}\left( \varepsilon \right) y_{i}\left( x\right) =o\left( \phi _{i}\left( \varepsilon \right) \right) \end{aligned}$$

is satisfied. And *generalized asymptotic expansions* are defined as

$$\begin{aligned} y\left( x,\varepsilon \right) =\sum \limits _{i=0}^{n}\phi _{i}\left( \varepsilon \right) y_{i}\left( x,\varepsilon \right) =O_{S}\left( \phi _{i+1}\left( \varepsilon \right) \right) ,\quad \varepsilon \rightarrow 0. \end{aligned}$$

Interesting cases occur when the function $$y\left( x,\varepsilon \right)$$ is not regular in $$\Omega$$, so expansion (3) is uniformly valid only in a restricted region $$\Omega _{0}$$$$\in$$$$\Omega$$ that is called *outer region*. In this case, the asymptotic expansion that is valid in outer region is often called *outer expansion* and (3) can be given as

4 $$\begin{aligned} y_{a}(x,\varepsilon )=E_{0}\phi =\sum _{i=0}^{n}\phi _{i}^{\left( 0\right) }\left( \varepsilon \right) y_{i}^{(0)}(x), \end{aligned}$$

where $\phi _{i}^{\left( 0\right) }\left( \varepsilon \right)$ is an asymptotic sequence and the special operator $E_{0}$ called the *outer expansion operator* at a given order $\phi \left( \varepsilon \right) .$

On the other hand, we encounter with a singular perturbation problem and we must introduce boundary layer domains. Here, in the simplest case (we assume that the problem has boundary layer near the origin $x=0$), we introduce an *inner region* which can be formally denoted $\Omega _{1}=\Omega -\Omega _{0}$ and located near the origin. The boundary layer variable is ${\overline{x}}=\frac{x}{\xi (\varepsilon )}$, $\xi (\varepsilon )$ being the order of thickness of this boundary layer. If a regular expansion can be constructed in $\Omega _{1}$, we can write

5 $$\begin{aligned} y_{a}(x,\varepsilon )=E_{1}\phi =\sum _{i=0}^{n}\phi _{i}^{(1)}(\varepsilon )y_{i}^{(1)}({\overline{x}}). \end{aligned}$$

This *inner expansion operator*$E_{1}$ is defined in $\Omega _{1}$ at the same order $\phi \left( \varepsilon \right)$ as the outer expansion operator $E_{0};$ thus, $y\left( x,\varepsilon \right) -E_{1}y\left( x,\varepsilon \right) =o(\phi \left( \varepsilon \right) )$ and so

$$\begin{aligned} y_{a}(x,\varepsilon )=E_{0}y\left( x,\varepsilon \right) +E_{1}y\left( x,\varepsilon \right) -E_{1}E_{0}y\left( x,\varepsilon \right) \end{aligned}$$

is clearly uniformly valid approximation to order $\delta \left( \varepsilon \right)$ satisfying the *modified Van Dyke principle (MVDP)*$E_{1}E_{0}y\left( x,\varepsilon \right) =E_{0}E_{1}y\left( x,\varepsilon \right)$. This is the main idea underlying the method of matched asymptotic expansions (MMAE). We know that MMAE which has been designed for finding uniformly valid approximations to singularly perturbed boundary value problems is a powerful mathematical technique. It is based on finding two different approximations for different two regions, which are called as inner region (where the solution exhibits rapid changes) and outer region (which is far from the inner region). In the last step, these two approximations are matched using the limit process to obtain a uniformly valid approximation. For more details we refer the reader to O’Malley (1974), Bender and Orszag (1978), Kevorkian and Cole (1981), Eckhaus (1973), Eckhaus (1979), Lagerstrom (1988), Hinch (1991), Van Dyke (1975), Johnson (2006), Verhulst (2006), Mauss and Cousteix (2002), Cousteix and Mauss (2004), Cousteix and Mauss (2009), Cathalifaud et al. (2010) and Nayfeh (1973).

## Successive Complementary Expansion Method

Sometimes the matching procedure in MMAE can be tedious or impossible. Therefore, we wish to present and examine an efficient asymptotic method named as *successive complementary expansion method*, which is designed by French scientists J. Mauss and J. Cousteix in order to obtain uniformly valid approximations to the boundary layer problems occur in fluid mechanics in Mauss and Cousteix (2002). In SCEM, instead of finding two different approximations to match later, a uniformly valid approximation that exactly satisfies the boundary conditions is proposed at the first step. So, thanks to the SCEM we will not be in need of any matching procedure. We can not ignore the fact that SCEM is not the first method which does not require any matching procedure. For instance, the WKB method and the Method of Multiple-scale Analysis also do not require any matching procedure (Cousteix and Mauss 2004). But their applicabilities are restricted to some certain problems. The uniformly valid SCEM approximation is in the regular form given as follows

6 $$\begin{aligned} y_{n}^{scem}(x,{\overline{x}},\varepsilon )=\overset{n}{\underset{i=0}{\sum }} \delta _{i}(\varepsilon )\left[ y_{i}(x)+\Psi _{i}({\overline{x}})\right] , \end{aligned}$$

where $\delta _{i}\left( \varepsilon \right)$ is an asymptotic sequence and $\Psi _{i}({\overline{x}})$ are the complementary approximation functions that depends on ${\overline{x}}$. Functions $y_{i}(x)$ are the outer approximations that have been found by MMAE and they only depend on *x*, not also on $\varepsilon$. In its regular form, SCEM is equivalent to MMAE. If the functions $y_{i}(x)$ and $\Psi _{i}({\overline{x}})$ also depend on $\varepsilon$, the uniformly valid SCEM approximation is named as *generalized SCEM approximation* and given in the following form (Cousteix and Mauss 2007; Mauss and Cousteix 2002; Cousteix and Mauss 2009)

7 $$\begin{aligned} y_{ng}^{scem}(x,{\overline{x}},\varepsilon )=\overset{n}{\underset{i=0}{\sum }} \overline{\delta }_{i}(\varepsilon )\left[ \overline{y}_{i}(x,\varepsilon )+ \overline{\Psi }_{i}({\overline{x}},\varepsilon )\right] \end{aligned}$$

with

$$\begin{aligned} y_{ng}^{scem}(x,{\overline{x}},\varepsilon )=y_{n}^{scem}(x,{\overline{x}} ,\varepsilon )+o(\delta _{n+1}\left( \varepsilon \right) ). \end{aligned}$$

The sequence of order functions $\delta _{i}\left( \varepsilon \right)$ may or not be same with $\overline{\delta }_{i}\left( \varepsilon \right) .$ If only one-term SCEM approximation is desired, then one seeks a uniformly valid SCEM approximation in the form of

$$\begin{aligned} y_{0}^{scem}(x,{\overline{x}},\varepsilon )=y_{0}(x,\varepsilon )+\Psi _{0}({\overline{x}},\varepsilon ). \end{aligned}$$

To improve the accuracy of SCEM approximation, the first SCEM approximation can be iterated using (7). It means that successive complementary terms will be add to the approximation. To this end, second SCEM approximation will be sought in the form of

$$\begin{aligned} y_{1}^{scem}(x,{\overline{x}},\varepsilon )=y_{0}(x,\varepsilon )+\Psi _{0}({\overline{x}},\varepsilon )+\varepsilon \left( y_{1}(x,\varepsilon )+\Psi _{1}({\overline{x}},\varepsilon )\right) . \end{aligned}$$

In Cousteix and Mauss (2007), error estimates for first and second SCEM approximations are given as $\left| y-y_{0}^{scem}\right| <\varepsilon K_{0}$ and $\left| y-y_{1}^{scem}\right| <\varepsilon ^{2}K_{1},$ where $K_{0}$ and $K_{1}$ are positive constants independent of $\varepsilon$ and *y* is the exact solution of the problem.

## Numerical examples

In this section, we present four numerical experiments to show the efficiency and the robustness of the proposed method. All the numerical calculations are performed using Shampine et al. (2000).

### *Example 1*

Consider the singular perturbation problem

8 $$\begin{aligned} \varepsilon y^{\prime \prime }(x)+2y^{\prime }(x)+e^{y(x)}=0, \quad x \in (0,1) \end{aligned}$$

subject to boundary conditions

9 $$\begin{aligned} y(0)=0, ~y(1)=0. \end{aligned}$$

This problem has a boundary layer near the point $x=0$ and uniformly valid asymptotic approximation is given as follows in Bender and Orszag (1978)

10 $$\begin{aligned} y(x)=\ln \left( \frac{2}{x+1}\right) -(\ln 2)e^{\frac{-2x}{\varepsilon }}. \end{aligned}$$

To obtain SCEM approximation for $n=0$ (first-term approximation) of (8–9), we seek an approximation in the form of

11 $$\begin{aligned} y_{0}^{scem}(x,{\overline{x}},\varepsilon )=y_{0}(x,\varepsilon )+\Psi _{0}({\overline{x}},\varepsilon ). \end{aligned}$$

For $\varepsilon =0$, Eq. (8) yields

12 $$\begin{aligned} 2y^{\prime }(x)+e^{y(x)}=0 \end{aligned}$$

and we have the outer solution as follows

13 $$\begin{aligned} y_{0}(x,\varepsilon )=\ln \left( \frac{2}{x+1}\right) . \end{aligned}$$

Now we look for a uniformly valid SCEM approximation in the form of

14 $$\begin{aligned} y_{ng}^{scem}\left( x,{\overline{x}},\varepsilon \right) =\ln \left( \frac{2}{ x+1}\right) +\Psi _{0}\left( {\overline{x}},\varepsilon \right) +\sum \limits _{i=1}^{n}\varepsilon ^{n}\left[ y_{i}\left( x,\varepsilon \right) +\Psi _{i}\left( {\overline{x}},\varepsilon \right) \right] , \end{aligned}$$

where ${\overline{x}}=\frac{x}{\varepsilon }$. Substituting (14) into the Eq. (8) and balancing the terms with respect to the powers of parameter $\varepsilon$ yields

15 $$\begin{aligned} \varepsilon \left( \Psi _{0}+\ln \left( \frac{2}{x+1}\right) \right) ^{\prime \prime }+2\left( \Psi _{0}+\ln \left( \frac{2}{x+1}\right) \right) ^{\prime }+e^{\left( \Psi _{0}+\ln \left( \frac{2}{x+1}\right) \right) }=0 \end{aligned}$$

and consequently, applying the balancing procedure we have

16 $$\begin{aligned} \Psi _{0}^{\prime \prime }({\overline{x}},\varepsilon )+2\Psi _{0}^{\prime }({\overline{x}},\varepsilon )=0 \end{aligned}$$

with the boundary conditions

17 $$\begin{aligned} \Psi _{0}(0,\varepsilon )=-\ln (2),~ \Psi _{0} \left( \frac{1}{\varepsilon },\varepsilon \right) =0. \end{aligned}$$

Then,

18 $$\begin{aligned} y_{0g}^{scem}(x,{\overline{x}},\varepsilon )=\left[ \ln \left( \frac{2}{x+1} \right) +\Psi _{0}({\overline{x}},\varepsilon )\right] \end{aligned}$$

is the first term of the SCEM approximation.

**Table 1:** Numerical results of Example 1 for $\varepsilon =0.1$

*x*	UVA solution (Bender and Orszag 1978)	SCEM approximation	Absolute error
0.000	0.000000000000	0.000000000000	0.000000000000
0.001	0.012725733435	0.012725733463	2.828981493e(−11)
0.005	0.060974133872	0.060974134008	1.359571344e(−10)
0.010	0.115695936573	0.115695936832	2.589762846e(−10)
0.100	0.504029730749	0.504029731985	1.2353316147e(−9)
0.200	0.498130190310	0.498130191712	1.4025156036e(−9)
0.300	0.429064776009	0.429064777435	1.4251414492e(−9)
0.400	0.356442418964	0.356442420392	1.4282036109e(−9)
0.500	0.287650603618	0.287650605047	1.4286180016e(−9)
0.600	0.223139292470	0.223139293899	1.4286740679e(−9)
0.700	0.162518353125	0.162518354554	1.4286816729e(−9)
0.800	0.105360437654	0.105360439083	1.4286826582e(−9)
0.900	0.051293283830	0.051293285259	1.4286828040e(−9)
1.000	−0.000000001428	0.000000000000	1.4286828220e(−9)

**Table 2:** Numerical results of Example 1 for $\varepsilon =0.001$

*x*	UVA solution (Bender and Orszag 1978)	SCEM approximation	Absolute error
0.000	0.000000000000	0.000000000000	0.000000000000
0.001	0.598340410221	0.598340410221	0.000000000000
0.005	0.688128170215	0.688128170215	0.000000000000
0.010	0.683196848278	0.683196848278	0.000000000000
0.100	0.597837000755	0.597837000755	0.000000000000
0.200	0.510825623765	0.510825623765	0.000000000000
0.300	0.430782916092	0.430782916092	0.000000000000
0.400	0.356674943938	0.356674943938	0.000000000000
0.500	0.287682072451	0.287682072451	0.000000000000
0.600	0.223143551314	0.223143551314	0.000000000000
0.700	0.162518929497	0.162518929497	0.000000000000
0.800	0.105360515657	0.105360515657	0.000000000000
0.900	0.051293294387	0.051293294387	0.000000000000
1.000	0.000000000000	0.000000000000	0.000000000000

**Fig. 1:**
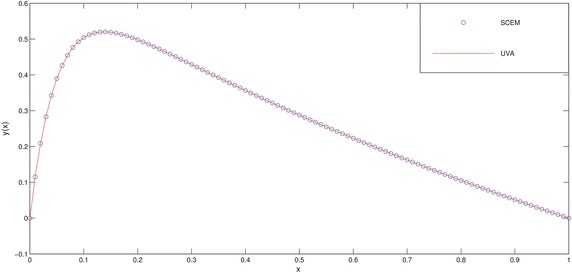
Comparison of SCEM and UVA solutions of Example 1 for $\varepsilon =0.1$

**Fig. 2:**
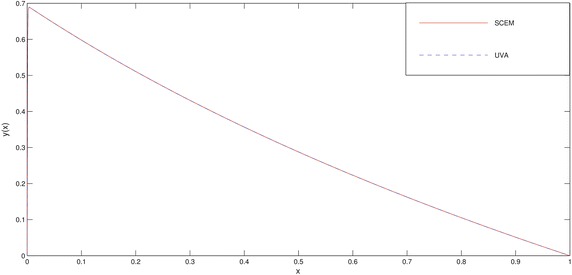
Comparison of SCEM and UVA solutions of Example 1 for $\varepsilon =0.001$

The numerical results of the problem are shown in Tables 1, 2 and in Figs. 1, 2. We deliberately choose the node points as possible as near the boundary layer (for this problem, near the point $x=0$) in order to see efficiency of the method.

### *Example 2*

Consider the second singular perturbation problem given in O’Malley (1974)

19 $$\begin{aligned} \varepsilon y^{\prime \prime }(x)+y(x)y^{\prime }(x)=0, \quad x\in \left( -1,1\right) \end{aligned}$$

subject to boundary conditions

20 $$\begin{aligned} y(-1)=0, \quad y(1)=-1, \end{aligned}$$

and with the exact solution

21 $$\begin{aligned} y(x,\varepsilon )=-\frac{1-e^{\frac{-(x+1)}{\varepsilon }}}{1+e^{\frac{-(x+1) }{\varepsilon }}}. \end{aligned}$$

This problem has boundary layer near the point $x=-1.$

**Table 3:** Numerical results of Example 2 for $\varepsilon =0.001$

*x*	Exact solution	SCEM approximation	Absolute error
−1.000	0.000000000000	0.000000000000	0.000000000000
−0.995	−0.986614298151	−0.986614102977	1.95174316e(−7)
−0.990	−0.999909204262	−0.999909184974	1.92884420e(−8)
−0.980	−0.999999995877	−0.999999995802	7.55888684e(−11)
−0.400	−1.000000000000	−1.000000000000	0.000000000000
−0.200	−1.000000000000	−1.000000000000	0.000000000000
0.000	−1.000000000000	−1.000000000000	0.000000000000
0.200	−1.000000000000	−1.000000000000	0.000000000000
0.400	−1.000000000000	−1.000000000000	0.000000000000
0.600	−1.000000000000	−1.000000000000	0.000000000000
0.800	−1.000000000000	−1.000000000000	0.000000000000
1.000	−1.000000000000	−1.000000000000	0.000000000000

**Fig. 3:**
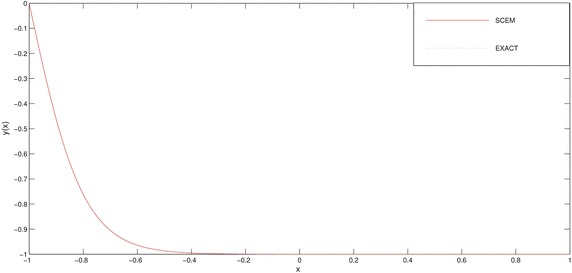
Comparison of SCEM and exact solutions of Example 2 for $\varepsilon =0.1$

**Fig. 4:**
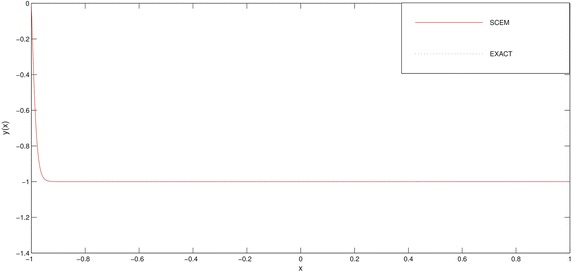
Comparison of SCEM and exact solutions of Example 2 for $\varepsilon =0.01$

In Table 3 and Figs. 3, 4 we deliberately choose the node points as possible as near the boundary layer (for this problem, near the point $x=-1$) in order to see efficiency of the method.

### *Example 3*

Consider the following singular perturbation problem given in O’Malley (1974)

22 $$\begin{aligned} \varepsilon y^{\prime \prime }(x)+e^{y(x)}y^{\prime }(x)-\frac{\pi }{2}\sin \left( \frac{\pi x}{2}\right) e^{2y(x)}=0, \quad x\in \left( 0,1\right) , \end{aligned}$$

with the boundary conditions

23 $$\begin{aligned} y(0)=0, \quad y(1)=0 \end{aligned}$$

and with the asymptotic solution

24 $$\begin{aligned} y=-\ln \left[ \left( 1+\cos \frac{\pi x}{2}\right) \left( 1-\frac{1}{2}e^{- \frac{x}{2\varepsilon }}\right) \right] +O\left( \varepsilon \right) . \end{aligned}$$

This problem has boundary layer near the point $x=0$.

**Table 4:** Numerical results of Example 3 for $\varepsilon =0.01$

*x*	Asymptotic solution (O’Malley 1974)	SCEM approximation	Absolute error
0.000	0.0000000000	0.0000000000	0.0000000000
0.001	−0.0476179808	−0.0476179344	4.633630e(−8)
0.005	−0.1998179193	−0.1998178062	1.130920e(−7)
0.010	−0.3317348800	−0.3317346943	1.857260e(−7)
0.050	−0.6496961369	−0.6496952868	8.501800e(−7)
0.100	−0.6835976643	−0.6835975658	9.854120e(−8)
0.200	−0.6683483288	−0.6683483281	6.84303e(−10)
0.300	−0.6371090868	−0.6371090868	4.61752e(−12)
0.400	−0.5927835996	−0.5927835996	3.10862e(−14)
0.500	−0.5347999967	−0.5347999967	2.22044e(−16)
0.600	−0.4623401221	−0.4623401221	1.11022e(−16)
0.700	−0.3743118452	−0.3743118452	0.0000000000
0.800	−0.2692764695	−0.2692764695	0.0000000000
0.900	−0.1453415344	−0.1453415344	0.0000000000
1.000	0.0000000000	0.0000000000	0.0000000000

**Table 5:** Numerical results of Example 3 for $\varepsilon =0.001$

*x*	Asymptotic solution (O’Malley 1974)	SCEM approximation	Absolute error
0.000	0.0000000000	0.0000000000	0.0000000000
0.001	−0.3317959489	−0.3317935405	2.40830e(−06)
0.005	−0.6512232379	−0.6512204646	2.77327e(−06)
0.010	−0.6897108336	−0.6897104246	4.08988e(−07)
0.050	−0.6916046583	−0.6916046583	2.66675e(−13)
0.100	−0.6869723256	−0.6869723256	0.0000000000
0.200	−0.6683710290	−0.6683710290	0.0000000000
0.300	−0.6371092397	−0.6371092397	0.0000000000
0.400	−0.5927836007	−0.5927836007	0.0000000000
0.500	−0.5347999967	−0.5347999967	0.0000000000
0.600	−0.4623401221	−0.4623401221	0.0000000000
0.700	−0.3743118452	−0.3743118452	0.0000000000
0.800	−0.2692764695	−0.2692764695	0.0000000000
0.900	−0.1453415344	−0.1453415344	0.0000000000
1.000	0.0000000000	0.0000000000	0.0000000000

**Fig. 5:**
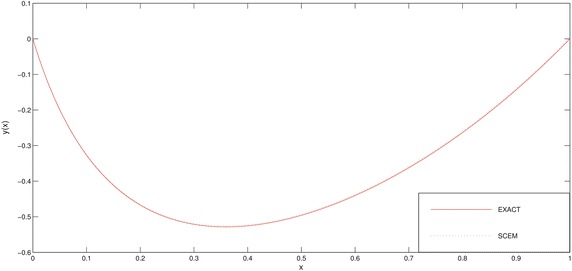
Comparison of SCEM and asymptotic solutions of Example 3 for $\varepsilon =0.1$

**Fig. 6:**
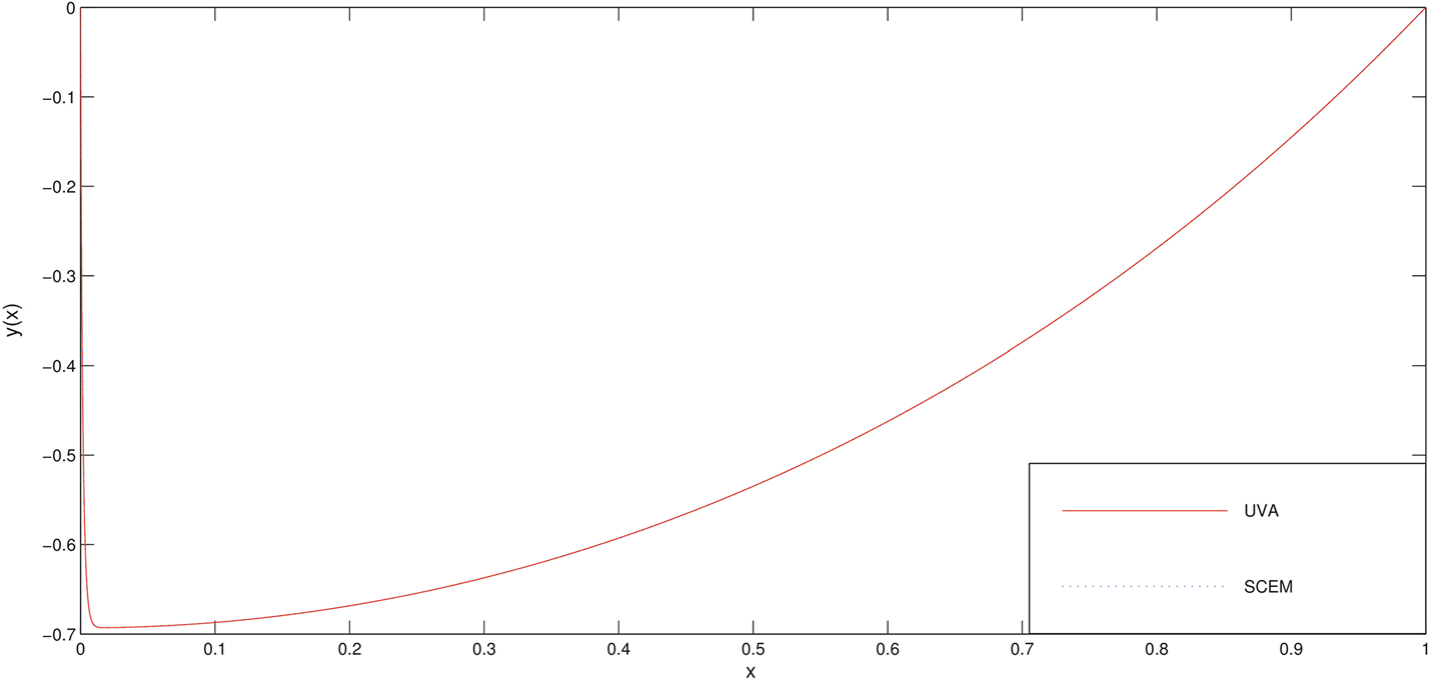
Comparison of SCEM and asymptotic solutions of Example 3 for $\varepsilon =0.01$

**Fig. 7:**
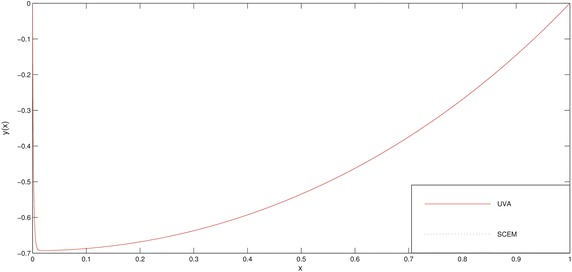
Comparison of SCEM and asymptotic solutions of Example 3 for $\varepsilon =0.001$

In Tables 4, 5 and Figs. 5, 6, 7 we deliberately choose the node points as possible as near the boundary layer (for this problem, near the point $x=0$) in order to see efficiency of the method.

### *Example 4*

As the last example, let us consider the singular perturbation problem given in Kevorkian and Cole (1981)

25 $$\begin{aligned} \varepsilon y^{\prime \prime }(x)+y(x)y^{\prime }(x)-y(x)=0, \quad x\in \left[ 0,1\right] \end{aligned}$$

with the boundary conditions

26 $$\begin{aligned} y(0)=-1,~ y(1)=3.9995. \end{aligned}$$

This problem has boundary layer near the point $x=0$ and the uniformly valid approximation is given in Kevorkian and Cole (1981) as

27 $$\begin{aligned} y=x+c_{1}\tanh \left( \left( \frac{c_{1}}{2}\right) \left( \frac{x}{ \varepsilon }+c_{2}\right) \right) , \end{aligned}$$

where $$c_{1}=2.9995$$ and $$c_{2}=\frac{1}{c_{1}}\ln \left[ \frac{c_{1}-1}{ c_{1}+1}\right]$$.

**Table 6:** Numerical results of Example 4 for $\varepsilon =0.01$

*x*	UVA solution	SCEM approximation	Absolute error
0.000	−1.0000000000000	−1.0000000000000	0.0000000000000
0.001	−0.5813960926361	−0.5791385346450	2.2575579e(−3)
0.005	1.1529592608432	1.1618243705484	8.8651097e(−3)
0.010	2.4659396723180	2.4727365388982	6.7968665e(−3)
0.050	3.0494963201391	3.0494968235068	5.0336773e(−7)
0.070	3.0694999908694	3.0694999930517	2.1823303e(−9)
0.100	3.0994999999988	3.0994999999993	4.689582e(−13)
0.200	3.1995000000000	3.1995000000000	0.000000000000
0.400	3.3995000000000	3.3995000000000	0.000000000000
0.600	3.5995000000000	3.5995000000000	0.000000000000
0.800	3.7995000000000	3.7995000000000	0.000000000000
1.000	3.9995000000000	3.9995000000000	0.000000000000

**Table 7:** Numerical results of Example 4 for $\varepsilon =0.001$

*x*	UVA solution	SCEM approximation	Absolute error
0.000	−1.0000000000000	−1.0000000000000	0.000000000000
0.001	2.4569396723180	2.4576445075493	7.0483523e(−4)
0.005	3.0044963201391	3.0044963670615	4.6922397e(−8)
0.010	3.0094999999988	3.0094999981336	1.8652617e(−9)
0.050	3.0495000000000	3.0495000000101	1.01216812e(−11)
0.070	3.0695000000000	3.0694999930517	7.23865412e(−13)
0.100	3.0995000000000	3.0995000000000	1.02140518e(−14)
0.200	3.1995000000000	3.1995000000000	0.000000000000
0.400	3.3995000000000	3.3995000000000	0.000000000000
0.600	3.5995000000000	3.5995000000000	0.000000000000
0.800	3.7995000000000	3.7995000000000	0.000000000000
1.000	3.9995000000000	3.9995000000000	0.000000000000

**Fig. 8:**
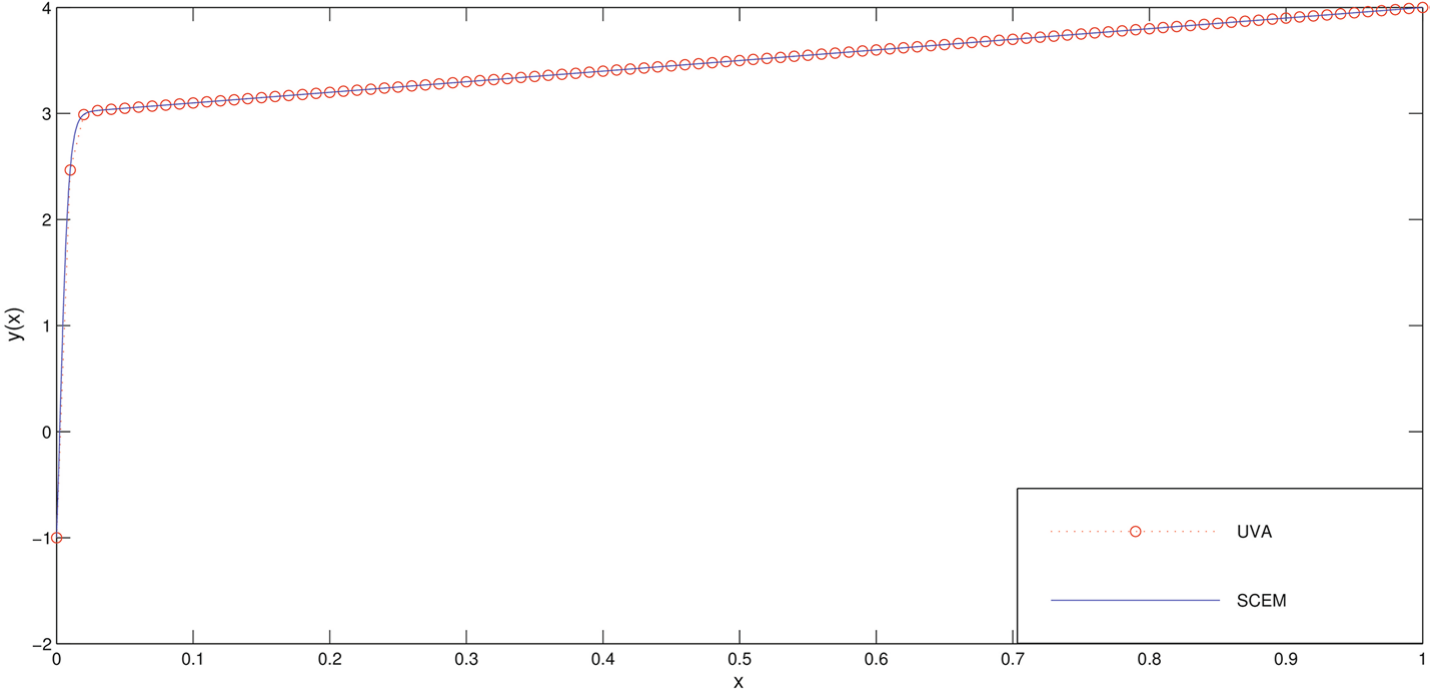
Comparison of SCEM and UVA solutions of Example 4 for $\varepsilon =0.01$

**Fig. 9:**
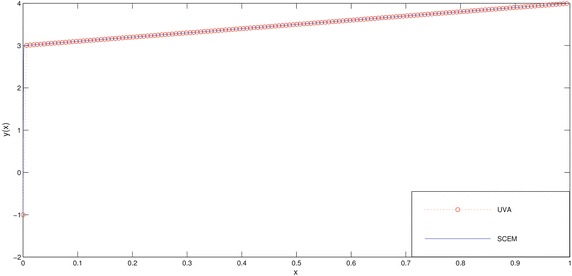
Comparison of SCEM and UVA solutions of Example 4 for $\varepsilon =0.001$

In Tables 6, 7 and Figs. 8, 9 we deliberately choose the node points as possible as near the boundary layer (for this problem, near the point $x=0$) in order to see efficiency of the method.

## Conclusion

In this paper, an efficient method so-called SCEM has been presented for singularly perturbed two-point second order nonlinear boundary value problems in ordinary differential equations and then the results have been compared with those which are previously obtained by various methods in literature. SCEM is very easy to implement using a mathematical software. As a result of our study, even though only one-term SCEM approximations are used in numerical examples, we obtain highly accurate approximations. As one can see in the numerical examples, SCEM does not require any matching procedures. Moreover, the boundary conditions are satisfied exactly, not asymptotically. Consequently, the present method is well-suited for solving nonlinear singular perturbation problems.
